# Molecular docking analysis of P2X7 receptor with the beta toxin from Clostridium perfringens

**DOI:** 10.6026/97320630016594

**Published:** 2020-08-31

**Authors:** Amit Kumar Solanki, Deepak Panwar, Himani Kaushik, Lalit C Garg

**Affiliations:** 1 National Institute of Immunology, New Delhi - 110067, India

**Keywords:** Clostridium perfringens beta-toxin, P2X7 receptor, protein 3D structure modeling, protein docking, protein-protein interaction

## Abstract

Clostridium perfringens beta-toxin (CPB) is linked to necrotic enteritis (over proliferation of bacteria) in several species showing cytotoxic effect on primary porcine endothelial and human precursor immune cells. P2X7 receptor on THP-1 cells is known to bind CPB. This is critical to understand the mechanism of pore formation for effective drug design. The structure of CPB and P2X7 receptor proteins were modeled using standard molecular modeling procedures (I-TASSER and Robetta server). This is followed by protein-protein docking (HADDOCK server) to study their molecular interaction. Interacting residues (19 residues from CPB and 21 residues from P2X7) were identified using the PISA server. Thus, we document the molecular docking analysis of P2X7 receptor with the beta toxin from Clostridium perfringens towards drug design and development of drugs to control necrotic enteritis.

## Background

C. perfringens type C strain causes lethal infections such as necrotic enteritis and enterotoxaemia in small bowel of cattle, sheep, goats and humans. The lethal disease can spread rapidly among unvaccinated herds causing huge economic losses to agriculture industry. Clostridium perfringens beta-toxin (CPB) is considered the major virulent factor and sufficient to reproduce the intestinal pathology associated with type C strain [1,2]. CPB belongs to the family of β-pore forming toxins and is known to share primary structure similarities with C. perfringens delta-toxin and Net-B toxin; S. aureus alpha-toxin, leukocidin, and gamma-toxin [3,4]. These toxins form cation-selective pores in target cell membrane (except delta-toxin) and induce swelling and lysis. The PFT- induced lysis of cells has been studied with several selective blockers. Tachykinin NK1 receptor antagonists, N-type Ca2+ channel blocker, bradykinin B2 receptor antagonists, necroststin-1and calpain inhibitors, significantly inhibited the CPB-induced leakage [5,6]. Previously it has been reported that alpha-toxin from S. aureus induced its cytotoxic effects through P2X7 receptor signaling and alpha-toxin induced hemolysin was inhibited by selective blockers of P2X1 and P2X7 receptors [7]. The P2X7 receptor, an extracellular ATP-gated ion channel highly expressed in immune effector cells. It has recently been implicated in CPB induced cell death. Selective P2X7 receptor antagonists significantly reduced CPB induced cytotoxicity in THP-1 cells [6, 8]. Thus, CPB like alpha-toxin uses specific proteinaceous receptor (P2X7) in lipid rafts for binding and oligomerization. However, the binding sites of P2X7 receptor and CPB are yet to be explored. Therefore, it is of interest to study molecular interaction of the beta toxin from C. perfringens with its receptor P2X7 by molecular docking. The amino acid residues involved in their interaction would be critical for CPB induced cytotoxicity and therefore, findings from this study may pave the way for designing and developing molecules to inhibit the interaction of CPB with the receptor and to control necrotic enteritis. Here using bioinformatics techniques, we deduced the 3D structures of CPB and P2X7, carried out molecular docking to identify their binding interface.

## Methodology

### Sequence data:

The complete amino acid sequences of CPB (309aa) and P2X7 (595aa) having accession number Q9L403 and Q99572 respectively were retrieved from the UniProt (http://www.uniprot.org/) database.

### Secondary structure analysis:

SOPMA (Self-Optimized Prediction Method with Alignment) was used to calculate the secondary structure features of CPB and P2X7 proteins [9].

### Binding sites assessment and protein docking:

Active sites in CPB and P2X7 models were identified using the CAST-p (http://sts.bioe.uic.edu/castp/) and COACH (http://zhanglab.ccmb.med.umich.edu/COACH/) servers [18-20]. CPB and P2X7 protein models were docked using the HADDOCK server [21- 24]. The models and complexes were visualized using PyMol (http://www.pymol.org; DeLano Scientific, San Carlos, CA, USA).

### Protein interaction interface analysis:

PISA (Protein Interfaces, Surfaces, and Assemblies) was used to analyze the protein-protein interactions and binding interface of CPB-P2X7 docked complex (http://www.ebi.ac.uk/pdbe/prot_int/pistart.html) [25]. It showed the presence of interacting residues elucidating extensive H-bonding interactions and interacting interface demonstrating the abundance of polar amino acid residues. Interactions energy of the generated CPB-P2X7 docked complex was also assessed using PISA.

### Structure modeling for CPB and P2X7:

The structures of CPB and P2X7 were modeled using threading and ab initio methods, respectively. The I-TASSER server (http://zhanglab.ccmb.med.umich.edu/I-TASSER) was used for the CPB protein structure prediction. A total of five models were generated by I-TASSER and the best model was selected on the basis of threading sequence identity and confidence score (C-Score) [10]. 3D Structure of P2X7 was predicted using the ab initio method employing the Robetta Server (http://robetta.bakerlab.org/) [11,12].

### Energy minimization and quality assessment:

Predicted models were subjected to energy minimization and refinement using ModRefiner [13]. Stereochemical properties in the models were assessed with Ramachandran plot using PROCHECK [14]. The coarse packing qualities and Ramachandran Z-scores of the refined structures were confirmed using the WHATIF server (http://swift.cmbi.ru.nl/servers/html/index.html) [15]. X-ray analysis, NMR spectroscopy and other theoretical calculations were verified using ProSA [16]. The models were further validated using the Protein Quality Predictor server (ProQ) [17].

## Results and Discussion:

CPB is the cause of necrotic enteritis in animals including pigs, goats and sheep causing huge financial loses to agriculture industry. Although the disease is treated with antibiotics regularly, such treatments are of little value as the disease progression is quite rapid and CPB once secreted is capable of producing enterotoxaemia independently of C. perfringens [1,2]. Also concerns have been raised against the large-scale use of antibiotics leading to emergence of microbial resistance [26,27]. Hence, the agriculture industry is in urgent need of effective treatment against CPB. Protein structure prediction has become an essential tool in structural biology towards the development of new drugs. The absence of crystal structure of CPB has hindered research activity in this field for quite some time now. In the past several selective inhibitors and antagonists of putative CPB receptors have been studied and tested in vitro but not employing silico approach [5,6]. Recent studies have implicated purinergic P2X7 receptor in CPB binding on THP-1 cells [8]. Hence, the 3D models and molecular docking studies of CPB and its receptor P2X7 offer better understanding of critical residues involved in binding, oligomerization and pore formation.

**Figure 1:**
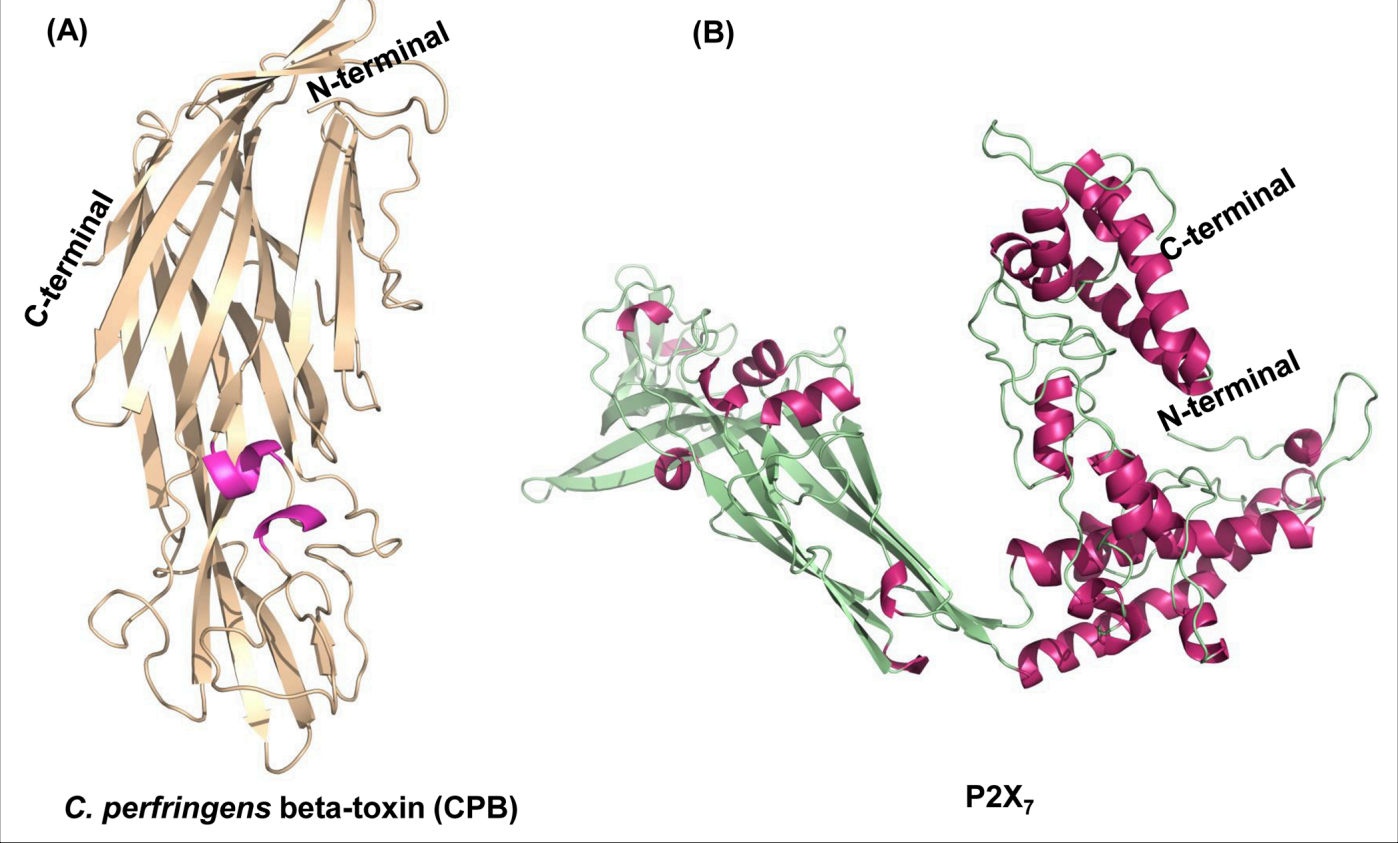
3D structure modeling of the CPB and P2X7 proteins. (A) The 3D CPB protein structure generated by I-TASSER. Magenta and wheat colors represent helix and beta sheets, respectively. (B) Predicted model of P2X7 by Robetta showing helix and beta sheets in pink and green colors, respectively. The N-terminus and C-terminus are marked.

In this direction, we generated 3D models of the CPB and P2X7. The 3D structures of CPB and P2X7 were ascertained on the basis of threading and ab-initio modeling methods, respectively. The tertiary structure of CPB generated using I-TASSER server had a confidence score (C-score) of -3.39 with TM score and RMSD value of 0.34 ± 0.11 and 14.2 ± 3.8Å, respectively. Additionally, the 3D structure of P2X7 was predicted employing Robetta server. Figure 1 shows the models of both CPB and P2X7. The CPB was found to have 19.09%, 31.39%, 10.03% and 39.48% of α helices, extended strands, β turns and random coils, respectively. P2X7 encompasses 25.88% α helices, 25.38% extended strand, 9.58% β turns and 39.16% random coils when calculated with SOPMA. Energy minimization of the two models to mimic the native confirmation using ModRefiner server resulted in energy minimized models with RMSD and TM-score of 0.178; 0.9992 and 0.607; 0.9951 for CPB and P2X7, respectively.

**Figure 2:**
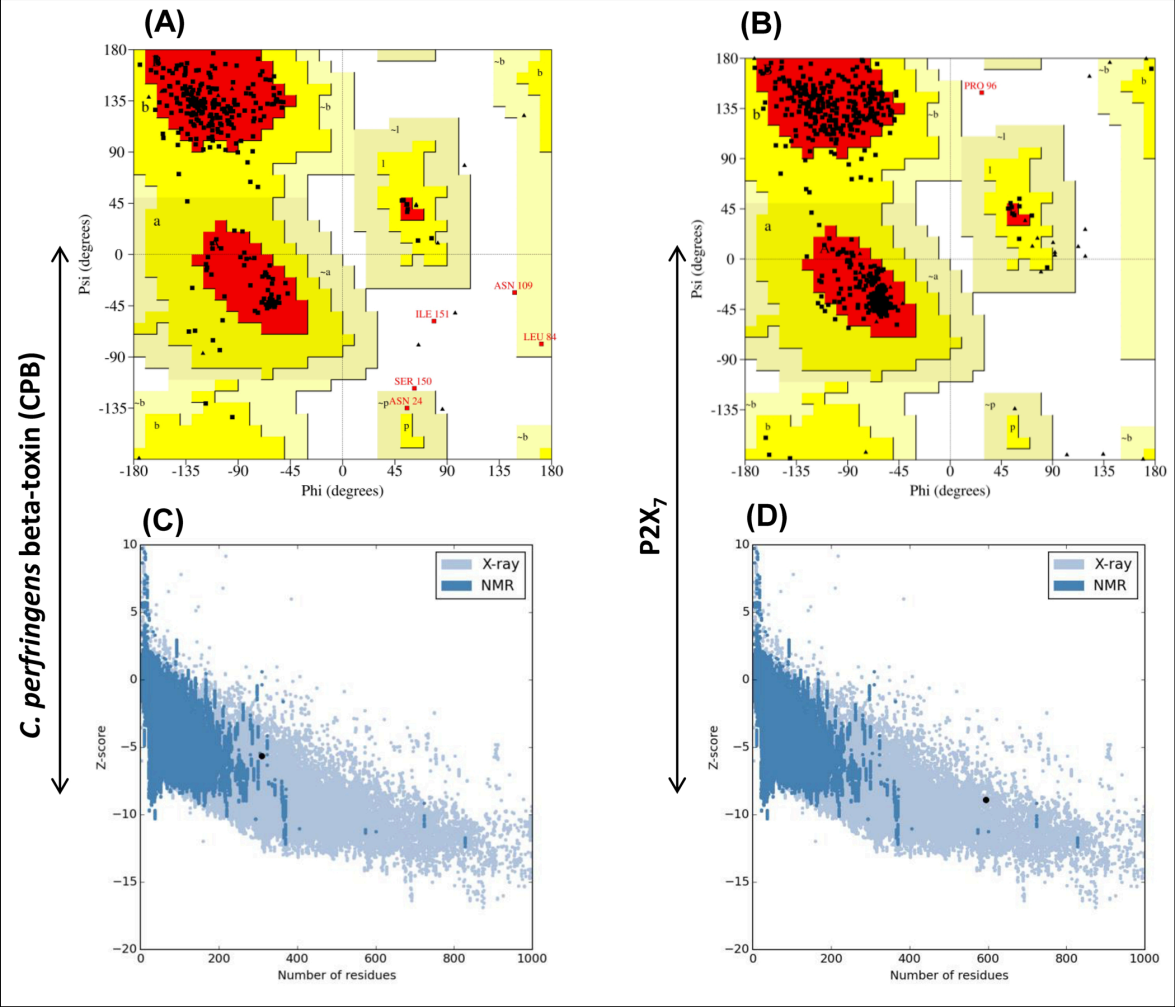
Ramachandran plot statistical analysis and ProSA Z-scores of CPB and P2X7 models. PROCHECK derived Ramachandran evaluation plots for CPB (A) and P2X7 (B) 3D structures. The black dots indicate the amino acids distributed in the red (most allowed) and yellow (allowed) regions. The predicted CPB (C) and P2X7 (D) Protein models had Z-scores (black point) of -5.63 and- 8.90, respectively.

**Figure 3:**
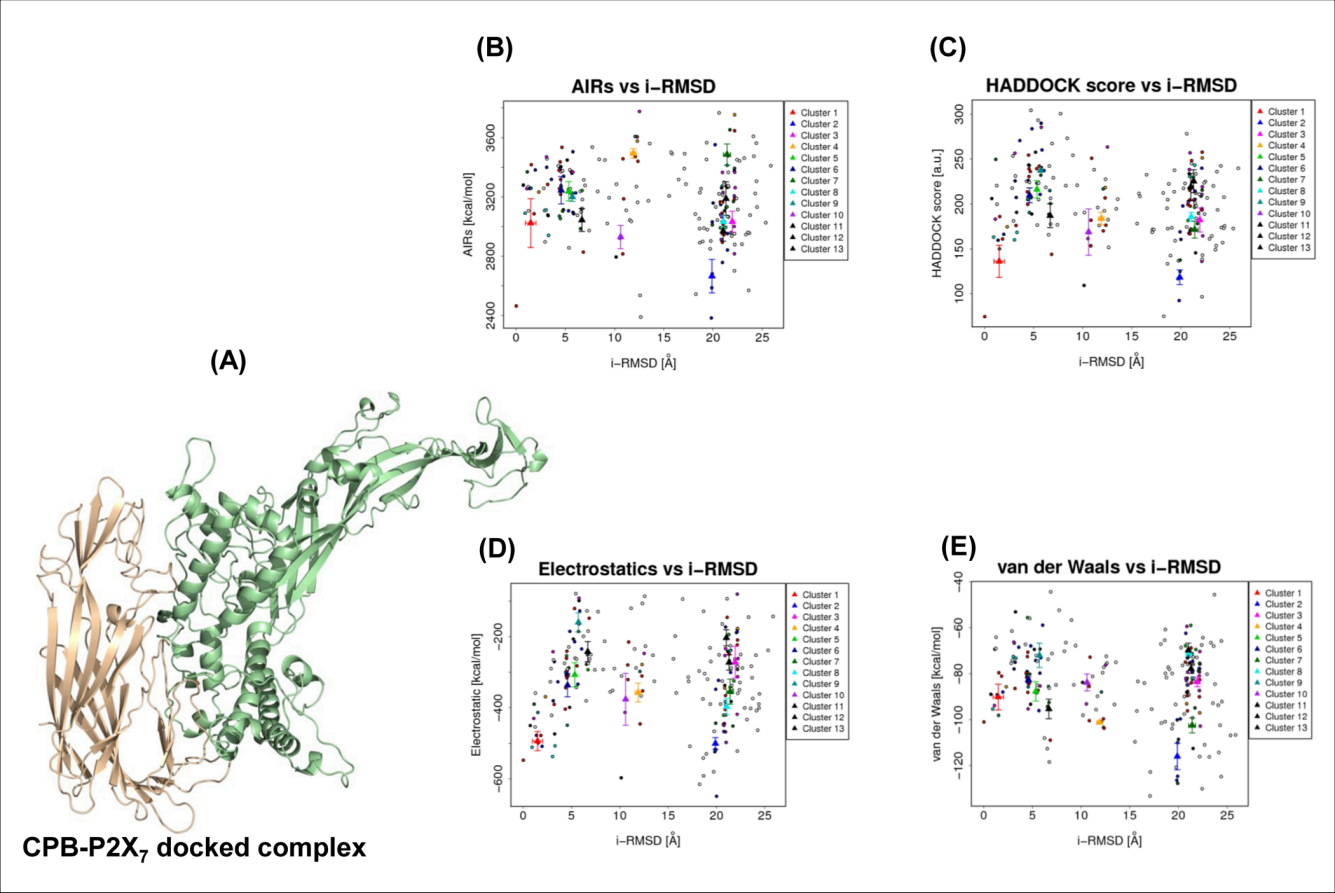
HADDOCK cluster analysis. (A) Selected CPB-P2X7 docked complex where CPB and P2X7 are shown in wheat and green, respectively. The HADDOCK docked models were plotted against their i-RMSDs; the color filled triangle corresponds to the individual cluster. (B) Interface-RMSDs (i-RMSDs) versus AIR energy (EAIR) plot for CPB-P2X7 complex model. The i-RMSDs were calculated on the backbone (CA, C, N, O, P) atoms of all residues involved in intermolecular contact using a 10Å cutoff. (C) The HADDOCK scores of clusters were plotted against their i-RMSDs. The HADDOCK score corresponds to the weighted sum of intermolecular electrostatic (D), van der Waals contacts (E), Desolvation, EAIR, and a buried surface area.

Geometric evaluations and stereochemical quality of the modeled 3D structures of CPB and P2X7 were performed using PROCHECK. Figure 2 A and B represents the Ramachandran plots calculating the distribution of phi and psi angles of the amino acid residues and classifies them in their respective quadrangle. Ramachandran plot analysis for the predicted CPB and P2X7 structures showed that 97.9% and 99.6% residues resided in the allowed regions, respectively. Whereas, 1.1% residues in CPB and 0.2% in P2X7 were present in the generously allowed regions while 1.1% of CPB and 0.2% of P2X7 amino acids resided in the disallowed regions, signifying the predicted models were reliable in terms of their backbone conformation. Furthermore, WHAT IF server assigned Ramachandran Z-scores of -0.390; 0.124 and structural average packing scores of -0.825; -1.219 for both CPB and P2X7 models, respectively. The models were analyzed for its fold reliability using ProSA server that estimated their energy profiles (Z-score) employing molecular mechanics force field. The Z-score predicts overall model quality and measures the cumulative energy deviation of the structure using random conformations. Figure 2 C and D shows the the quality score calculated by PRoSA for protein structures, wherein predicted Z-scores values were -5.63 for CPB and -8.90 for P2X7, evidencing highly reliable structures. Additionally, the energy plots showed the local model quality based on plotting energies as a function of amino acid sequence position. The quality of the protein structures was also validated using ProQ. The results showed that the predicted LG score of 4.414; 3.254 and MaxSub score of 0.179; 0.211 for both CPB and P2X7 models respectively suggested that protein models were in an acceptable range. These refined models were docked and best cluster representing CPB-P2X7 complex was selected and interacting residues were identified in CPB and P2X7 receptor. Residues corresponding to CPB and P2X7 proteins mentioned n Table 1 were subjected to protein docking. HADDOCK returned 108 structures in 13 cluster(s), which represents 54.0% of the water-refined models, analysis of best 10 clusters are given in Table 2. Figure 3 (A-E) shows the energy plots from 13 clusters, cluster 2 with HADDOCK score: 118.4 +/- 16.5 Kcal/mol, cluster size: 17, electrostatic energy: -500.6 +/- 33.1 Kcal/mol and Z-Score: -2.0 was selected as the best CPB-P2X7 docked complex for further study.

**Figure 4:**
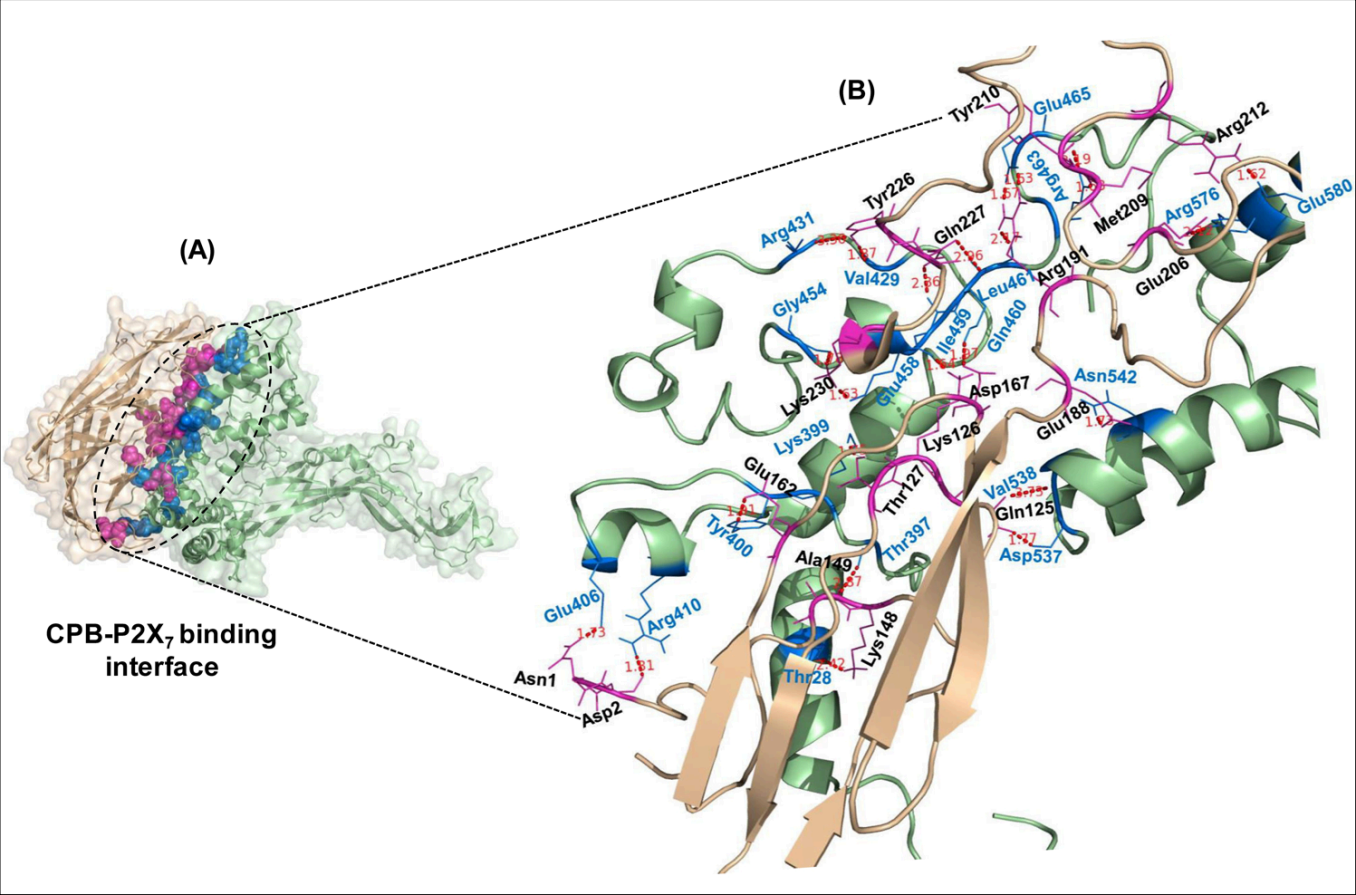
CPB-P2X7 interacting interface and binding residues. (A) Structural overview of CPB-P2X7 interacting interface predicted by PISA, the interacting residues are shown in spheres (CPB: magenta and P2X7: blue). (B) A close view of CPB-P2X7 binding interface showing the interacting residues corresponding to CPB and P2X7 proteins in magenta and blue, respectively. Dotted lines (red) represent atomic distances between hydrogen bonds formed by binding residues.

Figure 4 and Table 3 provides the intermolecular protein-protein interactions and surface interface areas of the docked complexes determined by the PISA server. CPB-P2X7 complex showed interaction having an interface area of 2314.8Å2 and solvation free energy (ΔiG) as -13.9 kcal/mol. Further analysis of the docked complex (CPB-P2X7) revealed the presence of interacting residues involved in extensive H-bonding and salt bridges are given in Table 4. The conserved CPB residues featuring in the complex can be exploited for designing effective drugs against CPB. In silico screening of chemical library to identify the compounds that would show favorable Van der Waals and electrostatic interactions with the binding site on the CPB or receptor may give a lead molecule(s) that would interfere with the binding of the CPB with its receptor P2X7 and negate subsequent effects of their interaction.

## Conclusion

The present study gives critical insight into CPB- P2X7 interaction and identification of interacting residues towards the design and development of drugs to control necrotic enteritis. The identified amino acid residues from CPB and P2X7 participating in protein-protein interaction can be targeted for effective drug design.

## Declaration on Publication Ethics:

The authors state that they adhere with COPE guidelines on publishing ethics as described elsewhere at https://publicationethics.org/. The authors also undertake that they are not associated with any other third party (governmental or non-governmental agencies) linking with any form of unethical issues connecting to this publication. The authors also declare that they are not withholding any information that is misleading to the publisher in regard to this article.

The authors are responsible for the content of this article. The Editorial and the publisher has taken reasonable steps to check the content of the article with reference to publishing ethics with adequate peer reviews deposited at PUBLONS.

## Figures and Tables

**Table 1:** Analysis of CASTp and COACH predictions for CPB and P2X7 protein binding sites

Binding site prediction: method used					
	CastP server		COACH server		
Protein	Cavity area (Å2)	Cavity volume		Predicted C-score	
		(cubic Å)	Protein		
CPB	291.9	360.8		COACH	Concavity
P2X7	5370.4	19192	CPB	0.41	0.15
			P2X7	0.21	0.32

**Table 2:** Statistical analysis for HADDOCK generated CPB and P2X7 docked complexes

S.No.	Cluster	HADDOCK scorea(a.u.)	Cluster Size	RMSD from overall lowest-energy structure (Å)	Vander Waals energy (Evdw) (kcal mol-1)	Electrostatic energyb(Eelec) (kcal mol-1)	Desolvation energy (Edesol) (kcal mol-1)	Restraints violation energy (kcal mol-1)	Buried surface area (Å2)	Z-Score
1	2	118.4 +/- 16.5	17	23.6 +/- 0.1	-116.0 +/- 11.6	-500.6 +/- 33.1	68.0 +/- 10.9	2665.4 +/- 226.88	4253.7 +/- 294.8	-2
2	1	136.1 +/- 35.7	26	2.0 +/- 1.6	-90.2 +/- 11.1	-493.9 +/- 53.7	22.7 +/- 12.5	3023.3 +/- 328.57	3022.0 +/- 178.1	-1.4
										
3	10	168.8 +/- 51.4	4	12.6 +/- 0.6	-83.8 +/- 7.3	-376.1 +/- 146.2	34.8 +/- 13.6	2929.6 +/- 156.28	3082.2 +/- 310.0	-0.3
4	7	171.6 +/- 19.1	6	26.2 +/- 0.6	-102.5 +/- 6.5	-354.5 +/- 70.5	-3.8 +/- 7.2	3486.8 +/- 143.55	3451.5 +/- 176.3	-0.2
5	3	182.2 +/- 26.9	11	24.7 +/- 0.2	-83.5 +/- 4.1	-272.0 +/- 90.0	16.8 +/- 12.2	3033.3 +/- 142.16	2565.7 +/- 77.1	0.2
6	4	183.8 +/- 13.9	7	14.0 +/- 0.7	-101.2 +/- 1.5	-357.3 +/- 53.2	7.0 +/- 4.3	3494.0 +/- 65.81	3363.0 +/- 115.6	0.3
7	8	185.7 +/- 10.1	6	24.9 +/- 0.1	-70.9 +/- 2.7	-396.7 +/- 45.4	32.7 +/- 9.5	3032.7 +/- 79.85	2623.6 +/- 199.6	0.3
8	11	187.0 +/- 26.5	4	8.9 +/- 0.1	-95.3 +/- 8.5	-242.1 +/- 56.5	26.3 +/- 16.1	3044.2 +/- 154.84	2850.7 +/- 123.7	0.4
9	6	209.7 +/- 16.6	7	6.0 +/- 0.0	-82.6 +/- 2.5	-336.5 +/- 65.5	35.1 +/- 4.1	3244.9 +/- 183.65	2619.9 +/- 217.6	1.2
10	5	216.2 +/- 19.4	7	7.2 +/- 0.4	-87.7 +/- 8.4	-307.2 +/- 67.8	41.5 +/- 8.6	3238.4 +/- 128.56	3165.4 +/- 125.4	1.4
a. The HADDOCK score = Evdw+ Eelec+ EAIR; In the equation, Evdw and Eelecrepresentvan der Waals and electrostatic energies, respectively. Whereas, EAIRindicates distance restraint contribution of AIRs. After the water refinement, the HADDOCK score was calculated as the following weighted sum: HADDOCK score = 1.0Evdw + 0.2Eelec + 1.0Edist + 0.1Esolv. Where, Esolv;solvationandEdist; distance restraints energies include both unambiguous interaction restraints and AIRs. b. Non-bonded interactions were calculated with the Optimized Potentials for Liquid Simulations (OPLS) force field using 8.5Å cut-off.

**Table 3:** PISA predicted CPB and P2X7 interacting interface

CPB			P2X7			CPB-P2X7 docked complex				
iNat	iNres	Surface (Å2)	iNat	iNres	Surface (Å2)	Interface area (Å2)	ΔiG (kcal/mol)	ΔiG P-value	NHB	NSB
243	62	16319	258	60	36488	2314.8	-13.9	0.821	24	10
iNat: indicates the number of interfacing atoms; iNres: indicates the number of interfacing residues ; Surface Å2: total solvent accessible surface area
Interface area: difference in the total accessible surface area of isolated and interfacing structures divided by 2 ; ΔiG: solvation free energy gain upon formation of the interface; ΔiG P-value: P-value of the observed solvation free energy gain ; NHB: number of hydrogen bonds; NSB: number of salt bridges

**Table 4:** PISA analysis of the H-bonding and salt-bridge interactions among the residues participating in CPB and P2X7 binding interface

Hydrogen Bonds			
S.No.	P2X7	Dist. [Å]	CPB
1	A:LYS 399[ HZ1]	1.75	B:THR 127[ OG1]
2	A:TYR 400[ HH ]	1.91	B:GLU 162[ OE1]
3	A:ARG 410[HH22]	1.81	B:ASP 2[ O ]
4	A:ARG 431[ N ]	3.36	B:TYR 226[ OH ]
5	A:GLN 460[HE22]	1.97	B:ASP 167[ OD1]
6	A:LEU 461[ N ]	2.96	B:GLN 227[ OE1]
7	A:ARG 463[HH22]	1.68	B:MET 209[ O ]
8	A:ARG 463[HH21]	2.19	B:TYR 210[ O ]
9	A:VAL 538[ N ]	3.73	B:GLN 125[ OE1]
10	A:ASN 542[HD21]	1.73	B:GLU 188[ OE2]
11	A:ARG 576[HH22]	2.12	B:GLU 206[ OE2]
12	A:THR 28[ OG1]	2.42	B:LYS 148[ HZ2]
13	A:THR 397[ O ]	2.87	B:ALA 149[ N ]
14	A:GLU 406[ OE1]	1.73	B:ASN 1[HD21]
15	A:VAL 429[ O ]	1.87	B:TYR 226[ HH ]
16	A:GLY 454[ O ]	1.78	B:LYS 230[ HZ3]
17	A:GLU 458[ OE2]	1.63	B:LYS 230[ HZ2]
18	A:GLU 458[ O ]	1.64	B:LYS 126[ HZ3]
19	A:ILE 459[ O ]	2.36	B:GLN 227[HE21]
20	A:LEU 461[ O ]	2.17	B:ARG 191[HH12]
21	A:GLU 465[ OE1]	1.67	B:ARG 191[HH11]
22	A:GLU 465[ OE2]	1.63	B:ARG 191[HH22]
23	A:ASP 537[ OD1]	1.77	B:GLN 125[HE22]
24	A:GLU 580[ OE1]	1.62	B:ARG 212[HH21]
Salt bridges			
S.No.	P2X7	Dist. [Å]	CPB
1	A:ARG 576[ NH1]	3.81	B:GLU 206[ OE2]
2	A:ARG 576[ NH2]	3.76	B:GLU 206[ OE1]
3	A:ARG 576[ NH2]	3.11	B:GLU 206[ OE2]
4	A:GLU 458[ OE2]	2.64	B:LYS 230[ NZ ]
5	A:GLU 465[ OE1]	2.67	B:ARG 191[ NH1]
6	A:GLU 465[ OE1]	3.35	B:ARG 191[ NH2]
7	A:GLU 465[ OE2]	3.59	B:ARG 191[ NH1]
8	A:GLU 465[ OE2]	2.64	B:ARG 191[ NH2]
9	A:GLU 580[ OE1]	3.34	B:ARG 212[ NH1]
10	A:GLU 580[ OE1]	2.63	B:ARG 212[ NH2]
